# Impacts of accreditation on the performance of primary health care centres: A systematic review

**DOI:** 10.51866/rv.274

**Published:** 2023-10-27

**Authors:** Jafar Sadegh Tabrizi, Atefeh As'habi, Maryam Nazari, Masoumeh Ebrahimi Tavani, Mehdi Haghi, Farid Gharibi

**Affiliations:** 1 BSc, MSc, PhD, Social Determinants of Health Research Centers, Semnan University of Medical Sciences, Semnan, Iran. Email: gharibihsa@gmail.com; 2 MD, PhD, Tabriz Health Services Management Research Center, Tabriz University of Medical Sciences, Tabriz, Iran.; 3 BSc, MSc, PhD, Food Safety Research Center (salt), Semnan University of Medical Sciences, Semnan, Iram.; 4 BSc, MSc, PhD, Food Safety Research Center (salt), Semnan University of Medical Sciences, Semnan, Iram.; 5 BSc, MSc, MPH, PhD, Quality Improvement, Monitoring and Evaluation Department, Center of Health Network Management, Deputy of Public Health, Ministry of Health and Medical Education, Tehran, Iran.; 6 BSc, MSc, PhD, Social Determinants of Health Research Center, School of Health and Nutrition, Lorestan University of Medical Sciences, Khorramabad, Iran.

**Keywords:** Accreditation, Primary health care, Performance

## Abstract

**Introduction:**

Evidence on the impacts of accreditation on primary health care (PHC) services is inconsistent. Thus, this study aimed to assess the impacts of accreditation on the performance of PHC centres.

**Method:**

This study systematically reviewed articles published from 2000 to 2019 in the Web of Science, Scopus, ScienceDirect, Springer, PubMed and ProQuest. The following keywords were used: ((primary care OR primary health care) AND (accreditation) AND (impact OR effect OR output OR outcome OR influence OR result OR consequences)). The database search yielded a total of41256 articles, among which 30 articles were finally included in the review.

**Results:**

Accreditation showed the most positive impacts on the quality, effectiveness, human resource management and strategic management of PHC services. Accreditation also positively affected safety, responsiveness, accessibility, customer satisfaction, documentation, leadership, efficiency and continuity of care. Few negative impacts were noted, including the possibility of accreditation being used as a bureaucratic tool, high cost of acquiring accreditation, difficulties in understanding the accreditation process, high staff turnover rate in accredited PHC centres and weak sustainability of some accreditation programmes.

**Conclusion:**

Given its numerous positive impacts, accreditation could be used to effectively improve the performance of PHC centres.

## Introduction

Primary health care (PHC) is an integral component of health care systems.1 It provides cost-effective services such as maternal and child, environmental, professional and mental health care; immunisation for communicable diseases; treatment of non-communicable diseases (NCDs); school hygiene; good nutrition; and health education and promotion. PHC centres serve as the initial point of contact between medical practitioners and the population.^1^ Evidence suggests that a well-developed health care system with misleading PHC networks could achieve better health outcomes.^2,3^ Accordingly, some countries have implemented health reforms aimed at strengthening their PHC systems in the last decades. Such reforms aim to control rising costs, with PHC services playing a central role in this aim and contributing to improving health equity.^2^

In the last few years, PHC services have encountered several challenges in the pursuit of improved quality and safety.^1,4^ Accreditation is one of the most known and applicable methods for assessing the performance of health care organisations (HCOs) and ensuring the quality and safety of health care service delivery.^5,6^ According to Rooney and Van Ostenberg, “accreditation is usually a voluntary programme, sponsored by a nongovernmental agency, in which trained external peer reviewers evaluate an HCO’s compliance with pre-established performance standards”.^7^ This process enables health care centres to benchmark themselves against top performers, making it one of the most influential systems for assessing and improving health care performance.^8,9^

The American College of Surgeons (ACS) was founded in 1913 with the objective of promoting hospital standardisation. It outlined specific membership prerequisites for surgeons and physicians, including the submission of medical documents regarding their professional competencies and preparation of patients’ records. In continuation of these efforts, the organisation established and implemented the Hospital Standardization Program in 1917. Finally, the ACS established the Joint Commission on Accreditation of Hospitals (JCAHO) in 1951 to meet the growing need for hospital accreditation.^10,11^ Surveys conducted during this period revealed that from 1951 to 1991, only eight accreditation programmes had been initiated. However, the number tripled in the next decade, especially in Europe.^12,13^ Yet, the implementation of accreditation in the PHC sector was delayed for a few decades. The Joint Commission International (JCI), which is the international branch of the JCAHO, published the first set of accreditation standards for PHC centres in 2008.^14^ Further, the Public Health Accreditation Board in the USA developed a set of standards for PHC accreditation in 2011, with the first public health organisation achieving accreditation in 2013.^15^

The hospital accreditation models used in Lebanon and Egypt have been recognised as the best and pioneering local accreditation models across the Eastern Mediterranean Region (EMR).^16^ In recent years, assessing and improving the quality of PHC services through accreditation have become a top priority in EMR countries.^17^ For example, Lebanon and Jordan initiated their PHC accreditation programmes in 2009, followed by Saudi Arabia in 2011 and Egypt in 2015, with technical assistance from the International Society for Quality in Healthcare (ISQua) and inspiration drawn from pioneering PHC accreditation models used in the USA and Canada. Similar programmes were also launched in other EMR countries such as Bahrain and Qatar.^18,19^

Several studies have evaluated the effectiveness of accreditation,^20-23^ but most of them have focused on hospital care.^24^ Accordingly, the understanding of the nature, acceptance and associated outcomes of accreditation in PHC settings is limited.^25^ In addition, the effectiveness of accreditation, especially in enhancing clinical performance, organisational processes and financial status, remains uncertain.^26,27^ Simultaneously, the use of accreditation in PHC settings is a relatively new concept, and its effectiveness, particularly in terms of improving the performance of PHC centres, is unclear.^15^

Given the inconsistent findings in the current literature regarding the impact of accreditation on PHC, further research is warranted.^28^ For instance, previous studies have indicated positive effects of accreditation, including improved quality of care, enhanced strategic planning, effective human resource management, better leadership, archiving and increased patient satisfaction.^29^ In contrast, some studies have highlighted negative impacts of accreditation, including high accreditation costs, substantial workload associated with the accreditation process and uncertainties of the benefits of accreditation.^25^ Accordingly, the current study aimed to assess the impacts of accreditation on the performance of PHC centres.

## Methods

### Study design and search strategy

This systematic review was conducted following the Preferred Reporting Items for Systematic Reviews and Meta-Analyses (PRISMA) protocol. Articles published from 2000 to 2019 were searched in the Web of Science, Scopus, ScienceDirect, Springer, PubMed and ProQuest. The following keywords were used: ((primary care OR primary health care) AND (accreditation) AND (impact OR effect OR output OR outcome OR influence OR result OR consequences)). In addition, grey literature was incorporated into the review to reduce the risks associated with publication bias.^30,31^ According to Pappas and Williams, ‘because of the delay between research and publication and because of the potential that some important research may never be published, access to innovative information is challenging. Grey literature is a tool to fill that void’.^32^ In total, 58 articles were identified from grey literature via searches in grey literature databases, a customised Google search engine and targeted websites such as ISQua, JCAHO and accreditation programme websites worldwide.^32-34^ The inclusion criterion was publication solely in the English language. The search strategy yielded a total of 41256 articles.

### Selection process

**Figure 1:**
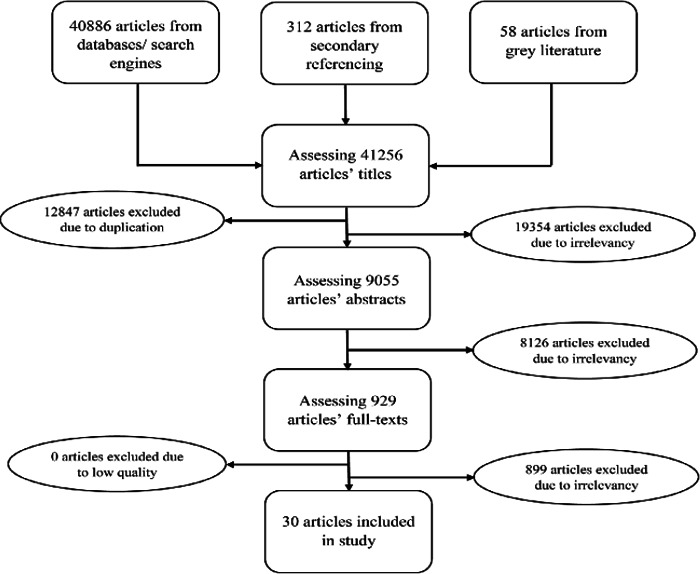
Flowchart of article selection for the systematic review.

The titles of all articles were reviewed. Initially, 12847 articles were excluded for duplication and 19354 articles for inconsistency with the study aim.^35^ Two senior researchers assessed 9055 abstracts, among which 8126 articles were excluded for irrelevance to the study aim. Thereafter, three senior researchers carefully assessed the full texts of 929 articles and excluded 899 articles. Finally, 30 articles were included in the review (Figure 1). To prevent the removal of related and useful articles, the researchers evaluated the articles in two independent groups. The article assessment lasted about 3 months.

### Quality and risk-of-bias assessments

The quality of the included articles was assessed. In particular, the Strengthening the Reporting of Observational Studies in Epidemiology, Consolidated Standards of Reporting Trials, PRISMA and Critical Appraisal Skills Programme were followed to appraise the quality of the cross-sectional, interventional, systematic review and qualitative articles, respectively. The responses for each item of the used tools were either ‘yes’ or ‘no’, which weighted 1 and 0, respectively. A ‘yes’ response indicated that the item was fulfilled, while a ‘no’ response indicated that the item was not fulfilled. Accordingly, the mean appraisal scores of the articles relative to the compliance to the protocol items were measured between 0 and 1 (as percentages). The scores were evaluated as follows: 0%-40% indicating a low quality; 41%–70%, moderate quality; and >70%, high quality.^36,37^ The articles that scored at least 70% were included in the analysis.^37^ The final included articles achieved an average of 89% compliance to their related quality appraisal tool. The senior researchers independently oversaw all review steps to minimise potential bias. Subsequently, the articles were unanimously selected.

### Data analysis and reporting

All articles were reviewed to evaluate the impacts of accreditation on the performance of PHC centres. All identified impacts of PHC accreditation were extracted, summarised and categorised. Finally, the identified impacts were categorised based on the affected performance indicators.

## Results

### Characteristics of the included studies

The analysis showed that only few studies investigated the impact of accreditation programmes on PHC services worldwide. The first related article was published in 2008, while the majority of the articles were published in 2018. Among the 30 selected studies, eight were conducted in low- and middle-income countries (LMICs), particularly in the EMR. These LMICs developed their accreditation programmes in recent years with the aid of organisations such as the ISQua and pioneering countries such as the USA and Canada, signifying that LMICs identified accreditation programmes as effective tools and, contrary to hospital accreditation, they embraced such programmes early.

### Contents of the included studies

The related contents of the included articles (positive and negative impacts of PHC accreditation programmes) are listed in Table 1.

**Table 1:** Identified impacts of accreditation on the performance of PHC centres.

Author(s)	Country (year)	Study design	Study objective	Positive impacts	Negative impacts
Paccioni et al.^38^	Canada (2008)	Mixed-method	To describe and understand the impact of the accreditation process on organisational control and quality management practices	- Quality: promoting and integrating quality, fostering personnel partnership in QI, measuring and analysing quality-related results - Effectiveness: enabling process orientation, standardising practice, expanding clinical supervision - Human resources: enabling socialisation of professionals, developing human resources, providing appropriate education and training, enhancing communication within institutions, establishing consultation mechanisms in self-assessment - Responsiveness: expressing expectations from professionals and other stakeholders - Strategic management: understanding the organisation and its values, enhancing value flexibility, enabling regular revision of action plans, facilitating professional cultural development, fostering cultural control - Leadership: developing flexible and facilitator leadership	- Facilitating bureaucratic actions - Enabling centralisation in decision-making - Decreasing the understanding of the accreditation process and its outcome among most staff
Al Tehewy et al.^39^	Egypt (2009)	Cross-sectional	To determine the impact of accreditation on patient and provider satisfaction and on compliance to some accreditation standards	- Quality: enhancing service quality aspects including waiting time, basic amenity and physical environment cleanness - Effectiveness: improving organisational performance - Human resources: increasing staff satisfaction - Customer satisfaction: increasing patient satisfaction	-
Szecsenyi et al.^40^	Germany (2011)	Interventional	To examine the effectiveness of the European Practice Assessment programme in improving management of PHC practices	- Quality: establishing quality-related policy, facilitating quality-related development, identifying quality-related problems - Safety: detecting safety concerns, analysing critical incidents - Customer satisfaction: facilitating complaint management	-
O’Beirne et al.^25^	Canada (2013)	Review/qualitative	To explore the current state of PHC accreditation	- Quality: enhancing quality assurance, quality culture and quality of delivered care - Effectiveness: assessing care processes, implementing effective performance programmes, emphasising cost-effectiveness by improving outcomes - Human resources: fostering organisational understanding, assessing staff performance, enabling teamwork - Safety: enhancing environmental safety, increasing awareness of staff about patient safety, facilitating risk management - Documentation: enabling clinical record audits - Accessibility: ensuring care accessibility - Efficiency: enhancing efficiency of care, reducing costs	
Abou Elnour et al.^41^	Australia (2014)	Qualitative	To explore surveyors’ perceptions regarding the impact of accreditation on patient safety and elicit suggestions for improving patient safety in general practices	- Quality: enabling policy-making related to QI - Effectiveness: enhancing effectiveness in clinical risk management - Human resources: implementing appointment systems, enhancing staff dedication in risk management - Safety: ensuring safety of general practice, physical environment, equipment and patients; facilitating clinical risk management system infection control; applying cold chain - Documentation: obtaining patient and electronic records, assessing clinical action and outcome - Accessibility: ensuring physical access to care	
Doorn-Klomberg et al.^42^	Netherlands (2014)	Comparative/observational	To examine the impact of accreditation on the quality of care among patients with diabetes, COPD and CVD	- Quality: enhancing the quality of diabetes care (foot examination, measurement of cholesterol levels, lipid-lowering medication prescription) and COPD care (spirometry performance, smoking cessation advice) - Effectiveness: improving cholesterol levels in patients with diabetes; reducing blood pressure, enabling smoking status registration and glucose measurement in patients with CVD	
El-Jardali et al.^29^	Lebanon (2014)	Mixed-method	To gain a better understanding of the impact of accreditation on the quality of care as perceived by PHC staff members and directors	- Quality: enabling resource allocation to QI programmes - Effectiveness: reinforcing quality standards, improving standards and delivery of health care - Human resources: facilitating staff involvement in QI, enabling human resource utilisation, increasing staff satisfaction, enabling staff involvement in the accreditation process, providing staff training and support to fulfil accreditation responsibilities, improving work conditions - Customer satisfaction: enabling complaint management, implementing customer satisfaction programmes, enhancing staff motivation and teamwork - Responsiveness: strengthening relationships with stakeholders, increasing responsiveness of health care organisations in change management - Documentation: enabling documentation especially in terms of quality - Strategic management: enabling strategic quality planning and priority setting - Leadership: providing clear vision of managers in improving quality	- Increasing workload and job stress in the accreditation process - Increasing accreditation costs
Yassoub et al.^43^	Lebanon (2014)	Qualitative	To assess the responsiveness of PHC centres to NCD and identify the needed health arrangements and responsibilities of PHC centres, the Ministry of Public Health and other health care system entities for PHC staff to purse a more preventive role against NCD	- Quality: enhancing the quality of delivered services, improving clinical practice - Effectiveness: enabling standardisation of delivered care and client-focused approach - Human resources: understanding quality and its requirements - Safety: ensuring staff and patient safety - Customer satisfaction: reducing patient complaints, enabling human resource management, increasing patients’ trust, enhancing teamwork, strengthening confidence in PHC services - Responsiveness: enabling community involvement, increasing responsiveness of PHC centres to the growing burden of NCD - Documentation: improving the quality of documentation - Strategic management: developing strategic plans, facilitating vision of staff, establishing comprehensive policies and procedures, developing strategy and objectives - Leadership: enabling pursuit of a leadership role - Accessibility: ensuring availability of specialists and medications - Efficiency: controlling NCD-related expenditures - Continuity of care: facilitating patient follow-up	
Nouwens et al.^44^	Netherlands (2014)	Randomised controlled trial	To determine the effectiveness of improvement plans in accreditation of PHC practices, focusing on cardiovascular risk management	- Quality/effectiveness: improving health service outcomes including smoking status, exercise control, diet control, registration of alcohol intake, measurement of waist circumference and fasting glucose level - Safety: facilitating CVD risk management	-
Diab^45^	Jordan (2015)	Cross-sectional	To assess the impacts of primary health accreditation standards on PHC and employee satisfaction in health care centres	- Quality: improving the quality of services, providing patient and family education - Effectiveness: providing patient care support - Human resources: facilitating staff management - Safety: implementing patient safety programmes - Responsiveness: meeting community health needs - Continuity of care: ensuring patient care continuum	
Ghareeb^46^	Qatar (2015)	Cross-sectional	To assess the changes resulting from the integration of Accreditation Canada International’s accreditation programme in PHC organisations	- Quality: facilitating quality management, obtaining quality-related results - Effectiveness: enabling organisational learning - Human resources: enabling human resource utilisation - Documentation: providing information, facilitating analysis - Strategic management: enabling strategic quality planning - Leadership: ensuring proper leadership	-
Harris et al.^47^	Canada (2015)	Mixed-method	To describe the impact of accreditation on the quality of delivered care in PHC services	- Human resources: enhancing interdisciplinary team functioning through enhancing team interactions and collaborations, understanding team members’ roles, increasing information, sharing resources	
Debono et al.^48^	Australia (2017)	Qualitative/interview	To examine stakeholders’ perspectives on general practice accreditation to identify programme strengths and weaknesses	- Quality: obtaining quality-related results - Human resources: facilitating peer review and collaborative learning - Safety: obtaining safety-related results - Accessibility: ensuring financial accessibility	
Alaradi^49^	Kuwait (2017)	Mixed-method	To assess the impact of accreditation of PHC centres in Kuwait from the perspective of health care professionals	- Effectiveness: boosting confidence in accreditation processes and results - Human resources: enabling employee participation in accreditation; enhancing teamwork, staff awareness and empowerment; obtaining staff opinions; removing professional barriers	Poor financial support and staff shortage and turnover impacting the sustainability of the accreditation programme
Shen et al.^50^	China (2018)	Comparative	To introduce the newly established registered dietitian accreditation systems in China	- Quality: promoting the quality of nutrition and dietetic profession - Accessibility: ensuring availability of dietitian services	
Nur Seha et al.^51^	Indonesia (2018)	Analytic observational and cross-sectional	To assess the impacts of accreditation on the job performance of electronic medical record clerks	- Human resources: enabling longer tenure and single task occupation - Documentation: ensuring completeness and accuracy of medical records	
Fu et al.^52^	Hong Kong (2018)	Retrospective	To assess the impact of accreditation on the obesity rate among students	- Quality: facilitating quality-based initiatives - Effectiveness: significantly decreasing the obesity rate among students	
Heffernan et al.^53^	USA (2018)	Case-control	To identify the benefits of participating in a public health accreditation programme	- Quality: focusing on QI efforts and initiatives - Strategic management: increasing awareness of organisational strengths/weaknesses	
Ingram et al.^54^	USA (2018)	Longitudinal repeated measures	To investigate the impact of the Public Health Accreditation Board on the delivery of PHC services	- Quality: improving the quality of the delivery of PHC services - Responsiveness: delivering core PHC services - Accessibility: enabling contribution of local health departments in core services	
Bialek^55^	USA (2018)	Descriptive	To assess the impact of public health department accreditation on workforce development in the USA	- Quality: providing quality training courses, reinforcing continuous improvement - Effectiveness: facilitating performance management and organisation-wide capacity building - Human resources: implementing workforce development activities, enabling the development of new skills and competencies - Responsiveness: implementing community assessment activities - Strategic management: enabling strategic planning, fostering organisational culture	
Beitsch et al.^56^	USA (2018)	Cross-sectional and longitudinal	To examine whether applying for Public Health Accreditation Board accreditation affects perceptions and activities regarding QI and performance management in local health departments	- Quality: implementing QI initiatives, enabling engagement in QI programmes - Effectiveness: enabling performance management, assessing outcome measures, improving effectiveness - Efficiency: ensuring efficiency and cost-saving	
Thomson et al.^57^	Tanzania (2018)	Cross-sectional	To explore variations in malaria-related knowledge and practices of drug retailers in ADDO and non-ADDO regions	- Quality: improving prescription of medication - Human resources: enhancing knowledge about anti-malaria medications - Accessibility: ensuring financial accessibility owing to lower drug prices	- Impacting regulation and increasing staff turnover in ADDO regions - Decreasing availability of malaria diagnostics in ADDO regions
Ishcomer et al.^58^	USA (2018)	Descriptive	To assess the impact of accreditation on collaborative partnerships in PHC centres	- Quality: facilitating QI efforts, fostering quality culture, enabling coordination of services - Effectiveness: fostering inter- and intradisciplinary partnerships, enabling health centre collaboration to share lessons learnt and best practices, facilitating performance management - Responsiveness: strengthening relationships with key partners, building social capital, reinforcing community resilience, leveraging resources and assets - Strategic management: facilitating comprehensive planning, enhancing the capacity to identify and address health priorities	
Ye et al.^59^	USA (2018)	Descriptive	To examine the impacts of accreditation on staff’s perceptions regarding workplace environment and job satisfaction	- Human resources: enhancing workplace environment, facilitating employee engagement, providing supervisory and organisational support, increasing overall job satisfaction and morale	
Siegfried et al.^60^	USA (2018)	Descriptive	To identify the QI and performance management benefits reported by public health departments as a result of participating in accreditation	- Quality: increasing awareness and focus on QI efforts, fostering QI culture, benchmarking QI - Effectiveness: enabling performance management, using information from QI processes in decision-making, obtaining effectiveness-related results - Human resources: facilitating QI training programmes among staff - Strategic management: implementing QI strategies and other strategies to evaluate effectiveness and quality - Efficiency: obtaining efficiency-related results	
Kittle & Liss- Levinson^61^	USA (2018)	Descriptive	To assess the benefits of accreditation in PHC centres	- Quality: facilitating QI efforts, fostering QI culture - Effectiveness: implementing performance improvement activity, enabling collaboration across departments within the agency	
Kronstadt et al.^62^	USA (2018)	Descriptive	To assess the benefits of accreditation in PHC centres	- Quality: implementing QI activities - Effectiveness: facilitating performance management, enabling partnerships - Human resources: increasing job satisfaction - Strategic management: enabling strategic planning - Leadership: providing future directions	
Moe et al.^63^	Canada (2019)	Descriptive	To examine the impact of accreditation as a QI strategy for community-based/fee-for-service family practices	- Quality: facilitating QI initiatives and formal recognition of excellence - Human resources: enabling human resource management - Efficiency: enabling logical cost-saving	
Brugueras et al.^64^	Spain (2019)	Observational descriptive	To evaluate the impact of accreditation of tuberculosis units	- Quality: improving quality of care - Effectiveness: enabling management of resistance, coordination with other departments and contact tracing	
Yeager et al.^65^	USA (2019)	Cross-sectional	To assess the impacts of accreditation on training needs, job satisfaction and awareness of public health concepts	- Human resources: facilitating job assessment, reducing skill gaps among staff, increasing awareness of staff about QI and various public health concepts, increasing job satisfaction	

PHC, primary health care; COPD, chronic obstructive pulmonary disease; CVD, cardiovascular disease; NCD, non-communicable disease; ADDO, accredited drug dispensing outlet; QI, quality improvement

### Performance indicators affected by PHC accreditation and their related items

The accreditation programmes positively affected the performance of PHC centres in various domains including quality, effectiveness, human resource management, safety, customer satisfaction, responsiveness, documentation, strategic management, leadership, accessibility, efficiency and continuity of care (Table 1). The identified impacts of accreditation of PHC centres were categorised based on their performance indicators.

Quality was defined as “the degree of excellence, extent to which an organisation meets clients’ needs and exceeds their expectations”.^12^ Its subdomains included quality improvement planning and policies, clinical management services and process orienting, and their related items were promoting and integrating quality, waiting area and time, improved culture, clinical practice, practice standardisation and patient/family education.

Effectiveness was defined as ‘the degree to which services, interventions or actions are provided in accordance with current best practice in order to meet goals and achieve optimal results’.^12^ Its subdomains included community involvement, internal and external collaboration and provision of cost-effective services, and their related items were involvement of stakeholders, consultation mechanisms in self-assessment, communication within institutions, effective performance improvement programmes, collaboration partners in the health care system and strengthening confidence in PHC services.

Human resources were defined as ‘the management of personnel requirements of the organisation’.^12^ Its subdomains included organisational culture, staff training and staff satisfaction, and their related items were extracted items such as socialisation of professionals, human resource development, support for practices, appropriate education and training, professional cultural development, teamworking, appointment systems, human resource utilisation, staff satisfaction and work conditions.

Safety was defined as ‘the degree to which the potential risk and unintended results are avoided or minimised’.^12^ The subdomains included risk management planning, safety culture and safe resources, and their related items were analysis of critical incidents, staff dedication to risk management, environmental safety, prevention of falls, physical environment of general practice, equipment safety, staff awareness of patient safety and infection control.

Customer satisfaction “measured how products or services supplied by a company meet or surpass a customer’s expectation”.^12^ The subdomains included satisfaction improvement and complaint system, and their related items were patient and customer satisfaction, complaint management and staff satisfaction.

Responsiveness was defined as ‘the ability of the health system to fulfil the legitimate expectations of individuals in interactions with the health system’.^12^ The subdomains included community needs, stakeholder education and service delivery environment, and their related items were responsiveness of PHC centres to the growing burden of NCD, responsiveness of centres when changes are to be implemented, support to fulfil their accreditation responsibilities, patient and family education, cleanliness, waiting area, waiting time and appropriate patient education.

Documentation was defined as “a critical vehicle for conveying essential clinical information about each patient’s diagnosis, treatment and outcomes and for communication between clinicians and payers”.^12^ The subdomains included information requirements, purposeful medical records and provision of user-friendly indicators, and their related items were clinical record auditing, patient records, quality of documentation, information and analysis, production of documented outcomes and actions and clinical risk management documents.

Strategic management was defined as “the formulation and implementation of the major goals and initiatives taken by a company’s top management on behalf of owners, based on consideration of resources and an assessment of the internal and external environments in which the organisation competes”.^12^ The subdomains included situation analysis, organisational values and objectives and action plan, and their related items were the understanding of and learning about the organisation, internalisation of organisational values with greater flexibility, frequent amendment of the organisational action plan with strategic quality planning, evidence-based priority setting and comprehensive policies in PHC centres.

Leadership was defined as the ‘ability to provide direction and cope with change. It involves establishing a vision, developing strategies for producing the changes needed to implement the vision, aligning people and motivating and inspiring people to overcome obstacles’.^12^ The subdomains included organisational vision and organisational motivation system, and their related items were developing a flexible and facilitator leadership, pursuing a leadership role, increasing motivation of staff, encouraging all employees to participate in the development of quality objectives and perceiving a positive impact on all values associated with cultural control.

Accessibility was defined as the ‘ability of clients or potential clients to obtain required or available services when needed within an appropriate time’.^12^ The subdomains included identifying and eliminating accessibility obstacles, and their related items were assessing access to care, simplifying certain bureaucratic processes, ensuring physical access and improving the availability of specialists and medications.

Efficiency was defined as ‘the degree to which resources are brought together to achieve results with minimal waste, re-work and effort’.^12^ The subdomains included cost-saving programme and cost-efficiency improvement, and their related items were enhancing efficiency and reducing costs by improving outcomes and controlling NCD-related expenditures.

Continuity of care was defined as ‘the provision of coordinated services within and across programmes and organisations over time’.^12^ The subdomains included the process of care and referral system, and their related items were patient flow, patient care continuum, patient follow-up and referral system.

### Performance indicators more affected by PHC accreditation

Although the implementation of accreditation programmes in PHC centres yielded numerous positive effects on various health system performance indicators, the number of stars acquired from the performance indicators (Table 2, vertically) showed that quality, effectiveness, human resource management, strategic management, safety, responsiveness and accessibility received more positive impacts.

**Table 2:** Positive impacts of accreditation on the performance indicators of PHC centres.

Indicator / Article	Quality	Effectiveness	Human resource	Safety	Customer satisfaction	Responsiveness	Documentation	Strategic management	Leadership	Accessibility	Efficiency	Continuity of care
Paccioni et al (2008)	^*^	^*^	^*^	N/M	N/M	^*^	N/M	^*^	^*^	N/M	N/M	N/M
Al Tehewy et al (2009)	^*^	^*^	^*^	N/M	^*^	N/M	N/M	N/M	N/M	N/M	N/M	N/M
Szecsenyi et al (2011)	^*^	N/M	N/M	^*^	^*^	N/M	N/M	N/M	N/M	N/M	N/M	N/M
O'Beirne et al (2013)	^*^	^*^	^*^	^*^	N/M	N/M	^*^	N/M	N/M	^*^	^*^	N/M
Abou Elnour et al (2014)	^*^	^*^	^*^	^*^	N/M	N/M	^*^	N/M	N/M	^*^	N/M	N/M
Doorn-Klomberg et al (2014)	^*^	^*^	N/M	N/M	N/M	N/M	N/M	N/M	N/M	N/M	N/M	N/M
El-Jardali et al. (2014)	^*^	^*^	^*^	N/M	^*^	^*^	^*^	^*^	^*^	N/M	N/M	N/M
Yassoub et al. (2014)	^*^	^*^	^*^	^*^	^*^	^*^	^*^	^*^	^*^	^*^	^*^	^*^
Nouwens et al. (2014)	^*^	^*^	N/M	^*^	N/M	N/M	N/M	N/M	N/M	N/M	N/M	N/M
Diab (2015)	^*^	^*^	^*^	^*^	N/M	^*^	N/M	N/M	N/M	N/M	N/M	^*^
Ghareeb (2015)	^*^	^*^	^*^	N/M	N/M	N/M	^*^	^*^	^*^	N/M	N/M	N/M
Harris et al. (2015)	N/M	N/M	^*^	N/M	N/M	N/M	N/M	N/M	N/M	N/M	N/M	N/M
Debono et al. (2017)	^*^	N/M	^*^	^*^	N/M	N/M	N/M	N/M	N/M	^*^	N/M	N/M
Alaradi (2017)	N/M	^*^	^*^	N/M	N/M	N/M	N/M	N/M	N/M	N/M	N/M	N/M
Shen et al (2018)	^*^	N/M	N/M	N/M	N/M	N/M	N/M	N/M	N/M	^*^	N/M	N/M
Nur Seha et al. (2018)	N/M	N/M	^*^	N/M	N/M	N/M	^*^	N/M	N/M	N/M	N/M	N/M
Fu et al (2018)	^*^	^*^	N/M	N/M	N/M	N/M	N/M	N/M	N/M	N/M	N/M	N/M
Heffernan et al (2018)	^*^	N/M	N/M	N/M	N/M	N/M	N/M	^*^	N/M	N/M	N/M	N/M
Ingram et al. (2018)	^*^	N/M	N/M	N/M	N/M	^*^	N/M	N/M	N/M	^*^	N/M	N/M
Bialek (2018)	^*^	^*^	^*^	N/M	N/M	^*^	N/M	^*^	N/M	N/M	N/M	N/M
Beitsch et al. (2018)	^*^	^*^	N/M	N/M	N/M	N/M	N/M	N/M	N/M	N/M	^*^	N/M
Thomson et al (2018)	^*^	N/M	^*^	N/M	N/M	N/M	N/M	N/M	N/M	^*^	N/M	N/M
Ishcomer et al. (2018)	^*^	^*^	N/M	N/M	N/M	^*^	N/M	^*^	N/M	N/M	N/M	N/M
Ye et al (2018)	N/M	N/M	^*^	N/M	N/M	N/M	N/M	N/M	N/M	N/M	N/M	N/M
Siegfried et al. (2018)	^*^	^*^	^*^	N/M	N/M	N/M	N/M	^*^	N/M	N/M	^*^	N/M
Kittle & Liss- Levinson (2018)	^*^	^*^	N/M	N/M	N/M	N/M	N/M	N/M	N/M	N/M	N/M	N/M
Kronstadt et al. (2018)	^*^	^*^	^*^	N/M	N/M	N/M	N/M	^*^	^*^	N/M	N/M	N/M
Moe et al (2019)	^*^	N/M	^*^	N/M	N/M	N/M	N/M	N/M	N/M	N/M	^*^	N/M
Brugueras et al. (2019)	^*^	^*^	N/M	N/M	N/M	N/M	N/M	N/M	N/M	N/M	N/M	N/M
Valerie et al. (2019)	N/M	N/M	^*^	N/M	N/M	N/M	N/M	N/M	N/M	N/M	N/M	N/M

* Shows the positive impacts of accreditation on performance indicators N/M, not mentioned

### Health systems more affected by PHC accreditation

The number of acquired stars by the included studies (Table 2, horizontally) indicated that the positive impacts of accreditation were more pronounced in developing countries. This was particularly notable in the studies conducted in LMICs, especially in the EMR. For example, the studies by Yassoub et al., El-Jardali et al. and Ghareeb, which assessed the effects of accreditation in Lebanon and Qatar, revealed that the accreditation implementation in developing countries yielded more positive effects on various performance indicators than did other accreditation programmes, even in developed countries.

Although the study results highlighted several advantages of accreditation on the performance of PHC centres, some negative points were noted. In the reviewed articles, accreditation of PHC centres required substantial resources (money, workforce and time) and led to increased bureaucracy and centralisation in decision-making. Further, the accreditation process and its outcomes were not necessarily understood by most staff. Poor financial support and staff shortage and turnover impacted the sustainability of the programme. A high staff turnover rate and marked staff shortage in some accredited health centres were also identified as negative impacts of accreditation.

## Discussion

The study results showed that the implementation of accreditation programmes in PHC centres yielded numerous positive impacts on various performance indicators such as quality, effectiveness, human resource management, strategic management, safety, responsiveness, accessibility, customer satisfaction, documentation, leadership, efficiency and continuity of care. Based on this finding, it can be concluded that accreditation has a positive impact on a wide range of performance indicators provided that accreditation standards emphasise main performance indicators adequately and the execution process is properly developed and implemented.

Although accreditation had positive impacts on many indicators, its influence on quality- related indicators was greater than that on other performance indicators. This may be attributed to the fact that accreditation was traditionally designed to improve quality.^12,66^ The lesser impacts of accreditation on other performance indicators may be related to the lack of relevant standards addressing these key performance indicators within accreditation programmes. The inclusion of appropriate standards related to other performance indicators in PHC accreditation models can help foster continuous improvement in the performance of PHC centres.

While accreditation standards traditionally emphasised quality and safety improvement, an evaluation of pioneering and successful accreditation programmes both globally and in the EMR revealed that their accreditation programmes covered the main performance indicators including high-quality care, safe care, accessibility of care, community-oriented care, continuity of care, appropriate and eflective management, human resource management, information management and customer rights and satisfaction.^31^ Comparing this scientific evidence with the current study finding reveals that the inclusion of each performance indicator with proper related standards/measures could improve the health performance indicators among PHC centres.

Although accreditation proves to be an eflective tool in improving performance in various settings, its positive impacts are more pronounced in LMICs. Herein, the studies that assessed the impacts of accreditation in Lebanon and Qatar revealed that their accreditation programmes in developing countries yielded more positive impacts on various performance indicators than did other accreditation programmes even in LMICs.^1,43,46^ This finding might be related to the fact that accreditation model standards in LMICs focus on a variety of performance indicators compared with those in high-income countries (HICs).^67-70^ This may be related to the greater need to address all functional dimensions of PHC in LMICs than in HICs owing to their weaknesses in these dimensions.^71^ Further, the success of accreditation in LMICs could be linked to the limited utilisation of performance improvement tools prior to the implementation of accreditation.^16,72,73^ Notably, developing countries, especially those in the EMR, have experienced long delays in the adoption of hospital accreditation, making them the pioneers of PHC accreditation worldwide. Given that these countries started their PHC accreditation programmes with the technical help of experienced countries in the field such as the USA and Canada, their rapid success is not surprising.^31^

Accreditation programmes in LMICs have been developed through collaboration and technical support from organisations such as the ISQua and inspiration from pioneering accreditation programmes in OECD countries. Therefore, greater effectiveness and efficiency in these accreditation programmes are expected, benefitting from collective experiences and rectification of previous mistakes and obstacles.^67-69^ A part of this disparity could be attributed to the differences in health personnel’s understanding of quality.

The study results highlight some limitations of PHC accreditation programmes. One notable constraint is the need for substantial resources to perform the entire accreditation process. However, the outcomes are expected to offset the associated costs by preventing medical errors, increasing the quality of health services, increasing patient satisfaction and boosting the credibility of accredited health care centres.^12^ This can be viewed as a cost-saving process that concurrently improves efficiency.^12,25,43^ In addition, organisers and users of accreditation should be aware of its potential to introduce bureaucratic processes and resolve potential complications through process mapping and amendments with active involvement of staff.

In Denmark, hospital staff held a negative perspective on accreditation. They believed the hospital accreditation programme as contributing to bureaucracy, overdocumentation, over-staffing and undue focus on partial processes. This led to the abrogation of the country’s accreditation programme in 2015.^74^ This reflects the result of inappropriate development and implementation of accreditation and oversight of existing challenges in successfully implementing an accreditation programme.

Considering the few avoidable negative impacts and the numerous positive impacts of accreditation in PHC settings, it could be presumed that applying PHC accreditation programmes will enhance the performance of health care centres. Given the numerous deficiencies in performance indicators within many health systems, the development of evidence-based and well-designed PHC accreditation programmes could improve the performance of PHC centres, especially those in LMICs.^4,71^ This could lead to more effective responses to community needs and rectification of existing shortcomings, particularly in terms of quality.^17^

The main limitations of this study are the inclusion of few related studies and the lack of assessment of the impacts of accreditation on performance indicators in all PHC centres. Further, the study considered only articles published in English, which could introduce a bias by excluding findings in other major languages related to the accreditation process.

Based on the study findings, the research team suggests some implications for practice, including the following: expanding PHC accreditation programmes worldwide, especially in LMICs; using existing evidence, particularly the experiences of organisations such as the ISQua and pioneering accreditation programmes such as the JCI, in developing standards and processes; focusing on all functional indicators in health systems, such as quality and safety of standards and measures, to meet societal health needs; and facilitating continuous improvement of developed accreditation programmes based on their evaluation results, mainly from stakeholders’ perspectives.

## Conclusion

Accreditation yields the most positive impacts on the quality, effectiveness, human resource management and strategic management of PHC services. There are only few negative impacts observed such as the possibility of illogical documentation in health care centres and the high primary cost and substantial effort required for the accreditation process. Given its numerous positive impacts but few avoidable negative impacts, accreditation could be used to improve the performance of PHC services, akin to hospital care.

## Review protocol registration

The review protocol was registered in Tabriz Health Services Management Research Center (Approval ID: 1394.580).

## References

[1] El-Jardali F, Ammar W, Hemadeh R, Jamal D, Jaafar M (2013). Improving primary healthcare through accreditation: baseline assessment of readiness and challenges in Lebanese context.. Int JHealth Plann Mgmt..

[2] Macinko J, Starfield B, Shi L (2003). The contribution of primary care systems to health outcomes within organization for economic cooperation and development (OECD) countries, 1970-1998.. Health Serv Res..

[3] Starfield B, Shi L, Macinko J (2005). Contributions of primary care to health systems and health.. Milbank Quart..

[4] World Health Organization. (2008). The World Health Report..

[5] Jovanovic B (2005). Hospital accreditation as method for assessing quality in healthcare.. Arch Oncol..

[6] Gharibi F, Tabrizi JS (2018). Development of an accreditation model for health education and promotion programs in the Iranian primary healthcare system: a Delphi study.. Health PromotPerspect..

[7] Rooney AL, Van Ostenberg P (1999). Licensure, Accreditation, and Certification: Approaches to Health Services Quality..

[8] Nandraj S, Khot A, Menon S, Brugha R (2001). A stakeholder approach towards hospital accreditation in India.. Health Policy Plan..

[9] Heaton C (2000). External peer review in Europe: an overview from the ExPeRT project.. Inter J Qual Health Care..

[10] Robert MD, James S, Jack G (1987). A history of the joint commission on accreditation of hospitals.. J Am Med Assoc..

[11] Montagu D (2003). Accreditation and Other External Quality Assessment Systems for Health Care..

[12] Shaw CD (2004). Toolkit for Accreditation Programs.. Melbourne: The International Society for Quality in Health Care (ISQua).

[13] Tabrizi JS, Gharibi F (2011). Systematic survey of accreditation models for designing a national model.. Sci J Kurdistan Univer Med Sci..

[14] Joint Commission International. (2008). Joint Commission International Accreditation Standards for Primary Care Centers..

[15] Bender KW, Kronstadt JL, Wilcox R, Tilson HH (2014). Public health accreditation addresses issues facing the public health workforce.. Am J PrevMed..

[16] Tabrizi JS, Gharibi F, Ramezani M (2012). Development of a national accreditation model in specialized clinics of hospitals.. Hakim Res J..

[17] World Health Organization. (2003). Quality and Accreditation in Health Care Services: A Global Review..

[18] Shaw CD (2000). External quality mechanisms for health care: summary of the ExPeRT project on visitatie, accreditation, EFQM and ISO assessment in European Union countries.. Inter J Qual Health Care..

[19] Braithwaite J, Westbrook J, Pawsey M (2006). A prospective, multi-method, multidisciplinary, multi-level, collaborative, social organizational design for researching health sector accreditation.. BMC Health Serv Res..

[20] Ovretveit J, Bate P, Cleary P (2002). Quality collaborative: lessons from research.. BMJ..

[21] Pomey M, Contandriopoulos A, Francois P, Bertrand D (2004). Accreditation: a tool for organizational change in hospitals?. Int J Health Care Qual Assur..

[22] Shaw CD (2003). Evaluating accreditation.. Int J Qual Health Care..

[23] Walshe K, Wallace L, Freeman T, Latham L, Spurgeon P (2001). The external review of quality improvement in health care organizations: a qualitative study.. Inter J Qual Health Care..

[24] Alkhenizan A, Shaw C (2011). Impact of accreditation on the quality of healthcare services: a systematic review of the literature.. Ann Saudi Med..

[25] O'Beirne M, Zwicker K, Sterling PD, Lait J, Lee Robertson H, Oeike ND (2013). The status of accreditation in primary care.. Qual Prim Care..

[26] Greenfield D, Braithwaite J (2008). Health sector accreditation research: a systematic review.. Int J Qual Heal Care..

[27] Hinchcliff R, Greenfield D, Moldovan M (2012). Narrative synthesis of health service accreditation literature.. BMJ Qual Saf..

[28] Greenfield D, Braithwaite J (2009). Developing the evidence base for accreditation of healthcare organisations: a call for transparency and innovation.. Qual Saf Health Care..

[29] El-Jardali F, Hemadeh F, Jaafar M (2014). The impact of accreditation of primary healthcare centers: successes, challenges and policy implications as perceived by healthcare providers and directors in Lebanon.. BMC Health Serv Res..

[30] Ahmed I, Sutton AJ, Riley RD (2011). Assessment of publication bias, selection bias, and unavailable data in meta-analyses using individual participant data: a database survey.. BMJ..

[31] Tabrizi JS, Gharibi F (2019). Primary healthcare accreditation standards: a systematic review.. Inter J Health Care Qual Assur..

[32] Pappas C, Williams I (2011). Grey literature: its emerging importance.. J Hosp Librar..

[33] Adams J, Hillier-Brown FC, Moore HJ (2016). Searching and synthesising ‘grey literature' and ‘grey information' in public health: critical reflections on three case studies.. Syst Rev..

[34] Franks H, Hardiker NR, McGrath M, McQuarrie C (2012). Public health interventions and behaviour change: reviewing the grey literature.. Public Health..

[35] Ghavarskhar F, Matlabi H, Gharibi F (2018). A systematic review to compare residential care facilities for older people in developed countries: practical implementations for Iran.. Cogent Soc Sci..

[36] Ebadifard Azar F, Azami-Aghdash S, Pournaghi-Azar F (2017). Cost-effectiveness of lung cancer screening and treatment methods: a systematic review of systematic reviews.. BMC Health Serv Res..

[37] Gagnier JJ, Kellam PJ (2013). Reporting and methodological quality of systematic reviews in the orthopaedic literature.. J Bone Joint Surg Am..

[38] Paccioni A, Sicotte C, Champagne F (2008). Accreditation: a cultural control strategy.. Int J Health Care Qual Assur..

[39] Al Tehewy M, Salem B, Habil I, El Okda S (2009). Evaluation of accreditation program in non-governmental organizations' health units in Egypt: short-term outcomes.. Inter J Qual Health Care..

[40] Szecsenyi J, Campbell S, Broge B (2011). Effectiveness of a quality-improvement program in improving management of primary care practices.. Canadian Med Assoc J..

[41] Abou Elnour A, Hernan AL, Ford D (2014). Surveyors' perceptions of the impact of accreditation on patient safety in general practice.. Med J Australia..

[42] Doorn-Klomberg AL, Braspenning JC, Wolters RJ, Bouma M, Wensing M (2014). Effect of accreditation on the quality of chronic disease management: a comparative observational study.. BMC Family Pract..

[43] Yassoub R, Hashimi S, Awada S, El-Jardali F (2014). Responsiveness of Lebanon’s primary healthcare centers to non-communicable diseases and related healthcare needs.. Inter J Health Plan Manag..

[44] Nouwens E, Lieshout JV, Adang E, Bouma M, Braspenning J, Wensing M (2012). Effectiveness and efficiency of a practice accreditation program on cardiovascular risk management in primary care: study protocol of a clustered randomized trial.. Implement Sci..

[45] Diab SM (2015). The effect of primary health accreditation standards on the primary health care quality and employees satisfaction in the Jordanian health care centers.. Inter J Academic Res Business Soc Sci..

[46] Ghareeb A (2015). Examining the Impact of Accreditation on a Primary Healthcare Organization in Qatar..

[47] Harris SB, Green ME, Brown JB (2015). Impact of a quality improvement program on primary healthcare in Canada: a mixed-method evaluation.. Health Policy..

[48] Debono D, Greenfield D, Testa L (2017). Understanding stakeholders’ perspectives and experiences of general practice accreditation.. Health Policy..

[49] Alaradi LK (2017). Assessing the Impact of Healthcare Accreditation from the Perspective of Professionals’ in Primary Healthcare Centres: A Mixed Methods Case Study from Kuwait..

[50] Shen X, Tang W, Yu Z, Cai W (2018). The history and development of registered dietitian accreditation systems in China and other comparable countries.. Nutr Res..

[51] Nur Seha H, Tamtomo D, Sulaeman ES (2018). Does accreditation status affect job performance of the electronic medical record clerks at community health center?. J Health Policy Manag..

[52] Fu YCA, To KCY, Kwan KMA (2018). School accreditation scheme reduces childhood obesity in Hong Kong.. Global Health Promot..

[53] Heffernan M, Kennedy M, Siegfried A, Meit M (2018). Benefits and perceptions of public health accreditation among health departments not yet applying.. J Public Health Manag Pract..

[54] Ingram RC, Mays GP, Kussainov N (2018). Changes in local public health system performance before and after attainment of national accreditation standards.. J Public Health Manag Pract..

[55] Bialek R (2018). From talk to action: the impact of public health department accreditation on workforce development.. J Public Health Manag Pract..

[56] Beitsch LM, Kronstadt J, Robin N, Leep C (2018). Has voluntary public health accreditation impacted health department perceptions and activities in quality improvement and performance management?. J Public Health Manag Pract..

[57] Thomson R, Johanes B, Festo C (2018). An assessment of the malaria-related knowledge and practices of Tanzania’s drug retailers: exploring the impact of drug store accreditation.. BMC Health Serv Res..

[58] Ishcomer J, Hewlett Noël W, Coffman J (2018). Public health accreditation and collaborative partnerships.. J Public Health Manag Pract..

[59] Ye J, P Leep C, Kronstadt J (2018). Public health employees’ perception of workplace environment and job satisfaction: the role of local health departments’ engagement in accreditation.. J Public Health Manag Pract..

[60] Siegfried A, Heffernan M, Kennedy M, Meit M (2018). Quality improvement and performance management benefits of public health accreditation: national evaluation findings.. J Public Health Manag Pract..

[61] Kittle A, Liss-Levinson R (2018). State health agencies’ perceptions of the benefits of accreditation.. J Public Health Manag Pract..

[62] Kronstadt J, Bender K, Beitsch L (2018). The impact of public health department accreditation: 10 years of lessons learned.. J Public Health Manag Pract..

[63] Moe G, Wang KH, Kousonsavath S (2019). Accreditation: a quality improvement strategy for the community-based family practice.. Healthc Q..

[64] Brugueras S, Roldan L, Rodrigo T (2019). Organization of tuberculosis control in Spain: evaluation of a strategy aimed at promoting the accreditation of tuberculosis units.. Arch Bronconeumol..

[65] Yeager VA, Balio CP, Kronstadt J, Beitsch LM (2019). The relationship between health department accreditation and workforce satisfaction, retention, and training needs.. J Public Health Manag Pract..

[66] Tabrizi JS, Gharibi F, Pirahary S (2013). Developing of national accreditation model for rural health centers in Iran health system.. Iran J Public Health..

[67] Egypt Ministry of Health. (2017). Standards for primary healthcare units/centers..

[68] Lebanon Ministry of Health. (2017). Self-assessment for Lebanon primary care standards..

[69] Jordan Ministry of Health. (2017). HCAC primary health care accreditation standards..

[70] Saudi Arabia Ministry of Health: Central Board of Accreditation for Healthcare Institutions. (2017). Primary healthcare standards.

[71] World Health Organization. (2000). The World Health Report 2000 - Health Systems: Improving Performance..

[72] Gharibi F, Tabrizi JS, Eteraf Oskouei MA, Asghari Jafarabadi M (2014). Effective interventions on service quality improvement in a physiotherapy clinic.. Health Promot Perspect..

[73] Tabrizi JS, Somi MH, Asghari S, Asghari Jafarabadi M, Gharibi F, Alidoost A (2015). The technical quality of delivered care for people with inflammatory bowel disease in Tabriz gastroenterology clinics.. Health Promot Perspect..

[74] Hlers LH, Jenses MB, Simonsen KB, Rasmussen GS, Braithwaite J (2017). Attitude towards accreditation among hospital employees in Denmark: cross-sectional survey.. Int J QualHealth Care..

